# Understanding eye care access for autistic adults and families: A convergent mixed-methods study

**DOI:** 10.1177/13623613251371509

**Published:** 2025-09-20

**Authors:** Chris Edwards, Abigail MA Love, Ru Ying Cai, Paul Constable, Daniel C Love, Ketan Parmar, Emma Gowen, Vicki Gibbs

**Affiliations:** 1 Aspect (Autism Spectrum Australia), Australia; 2 Griffith University, Australia; 3 The University of Melbourne, Australia; 4 La Trobe University, Australia; 5 Flinders University, Australia; 6 University of Cincinnati, USA; 7 The University of Manchester, UK; 8 The University of Sydney, Australia

**Keywords:** autism, autism-friendly, clinical/diagnoses, eye care, healthcare, vision

## Abstract

Autistic people face persistent barriers to accessing healthcare, yet little is known about their experiences receiving eye care, despite elevated rates of vision conditions. This convergent mixed-methods study explored the eye care experiences of 127 autistic adults and 69 parents of autistic children living in Australia and Aotearoa New Zealand. Through an online survey incorporating both structured and open-ended items, we examined access patterns, perceived barriers and enablers, and unmet needs. Quantitative data revealed widespread challenges, including sensory discomfort, communication difficulties, and financial costs, while qualitative responses foregrounded how provider interaction, clinical pacing, and environmental conditions shaped overall experience. Although participants valued clearer communication and sensory-informed adjustments, these were described as insufficient in the face of structural barriers such as affordability and inaccessible service locations. These findings align with emerging models of autism-informed healthcare that advocate for systemic, rather than ad hoc, approaches to accessibility. Our results underscore the need for proactive, cross-sector solutions developed in partnership with autistic people, which embed neurodiversity-affirming principles into eye care design, delivery, and policy. Addressing these inequities is both an ethical and practical imperative if we are to ensure that eye care services are inclusive, sustainable, and responsive to diverse ways of being.

**Lay abstract:**

Autistic people often face barriers when using healthcare services, but little is known about their experiences with eye care. This is important because autistic people are more likely to have vision problems like needing glasses, having a lazy eye, or having trouble with how their eyes work together. In this study, we asked 127 autistic adults and 69 parents of autistic children in Australia and Aotearoa New Zealand about their experiences receiving eye care. People completed an online survey that included multiple-choice questions and space to describe their experiences in their own words. Many participants said that eye care could be stressful or confusing. Common challenges included unclear instructions, bright lights, noisy environments, feeling rushed, and staff not understanding autism. Some people avoided going to eye care professionals altogether because they could not afford glasses or found the environment too overwhelming. Participants said that small changes like using plain language, giving more time, and creating a calm environment helped make care more accessible. However, these small changes were not always enough, especially when services were too expensive or hard to get to. To improve access to eye care, changes need to happen at all levels, including how clinics are designed and how staff are trained. These changes should be made together with autistic people to make sure that services meet their needs and feel respectful and welcoming. This study shows the need to redesign eye care so that it works better for autistic people and their families.

Autistic people frequently encounter significant and multi-layered barriers when accessing healthcare, contributing to persistent disparities in health outcomes compared to non-autistic people (Babalola et al., 2024; Walsh et al., 2020; Weir et al., 2022). A growing body of research identifies communication breakdowns, sensory discomfort, and limited provider understanding as key challenges that undermine both access to care and its quality (Babalola et al., 2024; Calleja et al., 2020; Corden et al., 2022; Radev et al., 2024; Shaw et al., 2024; Walsh et al., 2020). These barriers often result in unmet health needs, heightened anxiety, and, for some, avoidance of healthcare altogether. While healthcare professionals report limited knowledge about autism and low self-efficacy in supporting autistic patients (Corden et al., 2022), the challenges within healthcare are not solely interpersonal. Rather, they reflect barriers operating across patient, provider, and systemic levels, highlighting the importance of implementing reasonable adjustments in communication practices, physical environments, and healthcare procedures (Babalola et al., 2024; Walsh et al., 2020).

One area of healthcare that remains underexamined in autism research is eye care. This gap is concerning, given the mounting evidence that autistic people experience significantly higher rates of ocular conditions, including amblyopia (significantly reduced vision in one eye), strabismus (misalignment of the eyes commonly known as a ‘crossed eyes’ or ‘squint’), and refractive errors (the need for glasses to see clearly), compared to non-autistic people (Little, 2018; Nadeem et al., 2024; Perna et al., 2023; Reynolds & Culican, 2023; Wu et al., 2023). These conditions often require early detection and/or sustained management, underscoring the need for accessible and autism-informed approaches to eye care.

Despite these elevated risks, autistic individuals’ eye care needs often go unmet. Studies have identified significant gaps in the receipt of comprehensive vision assessments among autistic children (Lindly et al., 2021; Swanson et al., 2020). However, targeted adjustments can make a difference. Research shows that with the use of visual aids, clear communication strategies, and sensory accommodations, most autistic children can successfully complete a standard eye examination (Coulter et al., 2015). Practical suggestions to further support access, such as familiarisation visits, reduced sensory demands, and flexible appointment pacing have also been outlined to support autistic individuals access eye care (Little, 2018).

Consistent with the broader trend of autism research that predominantly focusses on childhood (Mason et al., 2022; Pellicano et al., 2014), far less is known about the eye care experiences of autistic adults. To date, only one published study has explored this area in depth. Parmar and colleagues (2022) reported on two qualitative investigations conducted in the United Kingdom that examined barriers and enablers to eye care access for autistic adults. In the first study, focus groups with 18 autistic adults identified core challenges including anxiety triggered by unfamiliar environments, inadequate communication, overwhelming sensory stimuli (such as bright lights and loud sounds), and difficulties navigating interactions with multiple staff members in different environments. The second study trialled an adapted autism-friendly eye examination protocol based on these findings and conducted feedback interviews with 24 autistic adults. Participants reported that adjustments such as providing clear, step-by-step explanations, detailed pre-appointment information about what to expect, maintaining staff continuity, and being mindful of sensory challenges and patient comfort significantly improved their experience and engagement. Together, these studies provide initial evidence that relatively simple modifications can make eye care more accessible for autistic adults. More importantly, these suggestions are consistent with broader recommendations in the healthcare literature (e.g. Doherty et al., 2023; Hamdan & Bennett, 2024; O’Hagan et al., 2023), which emphasise that proactive modifications and accommodations are essential for ensuring healthcare is truly accessible and equitable.

## Current study

Despite these advances, no research to date has examined the experiences of autistic people accessing eye care in the Australasian context. Healthcare systems, funding models, and cultural norms differ between countries, making it critical to understand how autistic people in Australia and Aotearoa New Zealand navigate these services. Importantly, both countries operate under publicly funded universal healthcare systems which provide subsidised access to a range of healthcare services. This focus is also supported by the close alignment between the two countries’ eye care systems, including shared professional standards, similar scopes of practice for optometrists and ophthalmologists, and coordinated oversight through bodies such as The Royal Australian and New Zealand College of Ophthalmologists and parallel regulatory frameworks in both countries.

In this study, we defined eye care as including both routine eye examinations (e.g. vision assessments, prescription for glasses, contact lenses, and ocular health checks) and non-routine or specialist care (e.g. referral to ophthalmology for further investigation). By adopting this inclusive definition, we aimed to capture the full range of experiences autistic people encounter across vision-related services. To reflect both adult and family experiences, we included autistic adults and parents of autistic children, recognising that access barriers can emerge at different life stages. We surveyed autistic adults and parents of autistic people across Australia and Aotearoa New Zealand to explore how they access eye care and what supports or barriers they encounter. Using a mixed-methods approach, we aimed to identify key barriers, enablers, and unmet needs affecting access to eye care in the region, with the goal of informing more inclusive, autism-affirming eye care practices.

## Methods

### Participants

Participants were eligible for inclusion if they were autistic adults (professionally diagnosed or self-identified)^1^ or parents of autistic children of any age living in Australia or Aotearoa New Zealand. There were no exclusion criteria based on experience with eye care; participants were invited to take part regardless of whether they, or their child, had ever seen an eye care professional. This approach acknowledged that barriers to access and preconceptions may prevent some autistic people and families from engaging with eye care in the first place and that their perspectives were critical for understanding any unmet need.

A total of 240 survey responses were received. Thirty-six were excluded because they did not respond beyond the consent, three were removed because they were submitted from outside Australia or Aotearoa New Zealand, and five were excluded based on age (under 18). Although participants were invited to complete the survey more than once if they held dual roles (e.g. as both an autistic adult and parent of an autistic child), no instances of duplicate participation were identified based on IP address and location data. The final eligible sample comprised 196 participants (127 autistic adults and 69 parents) who provided informed consent. Of these, 161 completed the survey in full and had an average completion time of 14.42 minutes. An additional 35 participants submitted partial responses, completing on average 68% of the survey. Most participants lived in Australia (*n* = 180), with a smaller number residing in Aotearoa New Zealand (*n* = 16). A breakdown of participant demographics, covering autistic adults, parents, and the autistic children described by parents is provided in Table 1.

**Table 1.** Participant demographics (self- and parent-reported).

Characteristic	Autistic adults (self-report), *n* = 127	Parents/caregivers (self-report), *n* = 69	Autistic children (parent-report), *n* = 69
Mean age in years (range)	39.44 (19–73)	44.00 (23–74)	13.45 (4–45)
Gender			
Female	74 (60%)	62 (94%)	24 (36%)
Male	28 (23%)	3 (5%)	41 (62%)
Non-binary	18 (15%)	1 (2%)	1 (2%)
Other	3 (2%)	0 (0%)	0 (0%)
Rather not say	1 (1%)	0 (0%)	0 (0%)
Ethnicity^a^			
Aboriginal Australian	3 (3%)	4 (6%)	5 (8%)
Torres Strait Islander	0 (0%)	1 (2%)	2 (3%)
Māori	4 (3%)	3 (5%)	3 (5%)
Pacific Islander	1 (1%)	1 (2%)	1 (2%)
South Asian	1 (1%)	2 (3%)	2 (3%)
South East Asian	3 (3%)	3 (5%)	2 (3%)
Middle Eastern	0 (0%)	1 (2%)	2 (3%)
African	2 (2%)	2 (3%)	2 (3%)
Latin American/Hispanic	0 (0%)	1 (2%)	2 (3%)
European/White	101 (84%)	53 (80%)	55 (83%)
Mixed heritage	11 (9%)	5 (8%)	7 (11%)
Other	8 (7%)	4 (6%)	5 (8%)
Rather not say	3 (3%)	0 (0%)	0 (0%)
Relationship status			
Single	65 (53%)	17 (26%)	
In a de facto relationship	21 (17%)	7 (11%)	
Married	24 (20%)	39 (59%)	
Other	9 (7%)	3 (5%)	
Education			
High school	16 (13%)	6 (9%)	
Vocational education	22 (18%)	12 (18%)	
Higher education	76 (63%)	43 (65%)	
Other	5 (4%)	4 (6%)	
Employment			
Full-time	31 (25%)	20 (30%)	
Part-time/casual	41 (33%)	19 (29%)	
Self-employed	15 (12%)	10 (15%)	
Unemployed	24 (20%)	4 (6%)	
Other	5 (4%)	7 (11%)	
Autism diagnosis/identification			
Formal diagnosis	110 (89%)		66 (100%)
Self-identification	13 (11%)		0 (0%)
Co-occurring conditions^a,b^			
ADHD/ADD	67 (58%)		33 (50%)
Intellectual disability	1 (1%)		17 (26%)
Anxiety disorder	59 (51%)		32 (48%)
Mood disorder	40 (34%)		6 (9%)
Sleep disorder	21 (18%)		9 (14%)
Other	38 (33%)		21 (32%)
Rather not say	2 (2%)		0 (0%)
No co-occurring conditions	15 (13%)		15 (23%)

a Participants could select multiple options.

b Participants were asked to exclude eye conditions, as a separate question addressed this later in the survey.

### Procedure

This study employed a convergent mixed-methods design, in which quantitative and qualitative data were collected concurrently through a single online survey (Creswell & Plano Clark, 2018). The quantitative items provided contextual insight into participants’ experiences with eye care, while the qualitative responses enabled deeper exploration of the personal and systemic factors shaping those experiences.

The study received ethical approval from the Griffith University Human Research Ethics Committee (2024/706). Data collection took place between January and April 2025. Recruitment was conducted via social media posts and newsletters circulated by Aspect (Autism Spectrum Australia), as well as through collaborator networks and professional bodies, including Optometry Australia and The Royal Australian and New Zealand College of Ophthalmologists. All recruitment materials included information about the study’s purpose and a link to the survey, which was hosted on Qualtrics. Participants were first presented with a plain-language information sheet and consent form. Informed consent was obtained by continuing with the survey.

### Online survey

The survey (see supplemental material survey) was designed to explore the eye care experiences of autistic adults and parents or caregivers of autistic people living in Australia and Aotearoa New Zealand. Participants first completed an eligibility screen and were then directed to questions tailored to their perspective, as either an autistic adult or a parent/caregiver of an autistic person.

The survey comprised four core sections. The first established eligibility. The second and third collected demographic information, including age, gender, ethnicity, education, and employment (see Table 1). The fourth section gathered contextual information about the individual participants’ eye care needs (e.g. frequency of eye exams, types of providers accessed), along with perceived barriers to care and enablers of a positive experience. Response options for items on barriers and enablers were informed by established autism-friendly healthcare principles, such as predictability, sensory regulation, and clear communication (e.g. Doherty et al., 2023; Parmar et al., 2022). The section on barriers and enablers also included open-text responses inviting participants to describe their experiences in their own words and to suggest ways to improve eye care for autistic people. These qualitative data were drawn from 11 open-text items, including both open-ended questions and optional free-text fields accompanying structured response options, yielding 668 responses with an average length of 31 words.

Following recent guidance on data integrity in online autism research (Pellicano, Adams, et al., 2024), we took several proactive steps to mitigate the risk of fraudulent participation. These included using built-in Qualtrics security features (such as CAPTCHA verification and preventing multiple responses from the same IP address) and removing participation incentives that might attract financially motivated responses from non-genuine participants. Instead, participants were invited to nominate a charity to receive a small donation in recognition of their contribution to this community-led research.

### Data analysis

Quantitative data were analysed descriptively using SPSS (Version 30). Frequencies and percentages were calculated for categorical variables, including participant demographics, eye care experiences, and perceived barriers and enablers. These descriptive statistics provided a contextual overview of the sample and informed the interpretation of patterns identified in the qualitative responses. Responses to individual survey items were analysed at the group level, with autistic adults and parents reported separately where relevant.

Qualitative data were analysed using the six-phase approach of reflexive thematic analysis (Braun & Clarke, 2006; Braun et al., 2019) and managed in NVivo (Version 15). This approach was selected for its emphasis on the researcher’s active, situated role in meaning-making, consistent with the study’s constructionist epistemology and experiential orientation, which recognises participants as experts in their own lives. Initial coding was conducted by the first author, an autistic autism researcher with expertise in autism-friendly principles (Edwards et al., 2025) and lived experience accessing eye care services. In line with reflexive thematic analysis, coding reflected the researcher’s interpretive engagement with the data. A primarily deductive yet flexible analytic approach was used, informed by the study’s quantitative findings and the researcher’s prior work, while remaining open to new insights expressed in participants’ own words. Both semantic and latent codes were developed to capture surface-level content and underlying patterns of meaning. Themes and subthemes were shaped and refined through iterative, reflexive discussion with the wider multidisciplinary team, which included autistic and non-autistic researchers, family members of autistic people, and eye care professionals, to support analytical depth and ensure the interpretation reflected diverse forms of expertise.

## Results

### Eye care access and vision needs of autistic people

Autistic adults and parents reported diverse engagement with eye care services (Table 2). Approximately one-third of autistic adults (35%, *n* = 43) and 42% of children (*n* = 27) had annual eye exams, while 34% (*n* = 41) of autistic adults and 38% (*n* = 25) of parents indicated that there had been at least one occasion when they or their child required eye care but were unable to access it. Most participants had accessed an optometrist (97% of adults; 77% of parents), although fewer had seen ophthalmologists (35% of adults; 44% of parents) or orthoptists (4% of adults; 16% of parents). Refractive errors (50% of adults; 42% of parents) were the most reported vision condition, followed by amblyopia (9% of adults; 17% of parents) and strabismus (3% of adults; 17% of parents). Less common but clinically significant conditions, such as glaucoma (3% of adults; 2% of parents) and retinopathy of prematurity (2% of adults; 2% of parents), were also noted. Notably, 28% of autistic adults (*n* = 33) and 33% of children (*n* = 21) were reported to have no known eye conditions.

**Table 2.** Eye care characteristics.

Characteristic	Autistic adults (self-report)	Autistic children (parent-report)
Frequency of eye examination	*n* = 122	*n* = 65
Twice a year	1 (1%)	6 (9%)
Annually	43 (35%)	27 (42%)
Every 2 years	44 (36%)	9 (14%)
Less than every 2 years	27 (22%)	11 (17%)
Never	3 (2%)	5 (8%)
Other	4 (3%)	7 (11%)
Eye care professionals accessed^a^	*n* = 118	*n* = 64
Optometrist	114 (97%)	49 (77%)
Ophthalmologist	41 (35%)	28 (44%)
Orthoptist	5 (4%)	10 (16%)
Never seen an eye care professional	3 (3%)	5 (8%)
Not sure	0 (0%)	3 (5%)
Eye care services used previously^a^	*n* = 118	*n* = 64
Routine eye examination	108 (92%)	48 (75%)
Prescribed glasses or contact lenses	104 (88%)	42 (66%)
Management of eye conditions	37 (31%)	15 (23%)
Paediatric eye care services	11 (9%)	22 (34%)
Other	13 (11%)	9 (14%)
Emergency eye care	16 (14%)	5 (8%)
Vision therapy	4 (3%)	9 (14%)
Occlusion therapy	3 (3%)	8 (13%)
Low vision services	1 (1%)	4 (6%)
Diagnosed eye conditions^a^	*n* = 119	*n* = 64
Refractive errors	59 (50%)	27 (42%)
No known eye conditions	33 (28%)	21 (33%)
Other (e.g. Keratoconus, dry eyes)	22 (18%)	10 (16%)
Amblyopia	11 (9%)	11 (17%)
Strabismus	3 (3%)	11 (17%)
Glaucoma	3 (3%)	1 (2%)
Retinopathy of prematurity	2 (2%)	1 (2%)
Cataracts	1 (1%)	1 (2%)
Retinoblastoma	1 (1%)	1 (2%)

a Participants could select multiple options.

### Barriers and enablers to accessing eye care for autistic people

Participants reported a range of challenges and supports related to eye care, with particular emphasis on sensory sensitivities, communication barriers, and environmental factors. Among autistic adults, the most frequently reported difficulties during eye examinations were sensory sensitivities (83%) and anxiety or stress (65%), as well as discomfort in the sensory environment (63%) and pressure to make quick decisions (61%) when accessing services. Similarly, parents identified anxiety or stress (77%) and sensory sensitivities (65%) as the most prevalent challenges during examinations for their child, along with a lack of understanding from staff (49%) and an uncomfortable sensory environment (46%) when navigating services. Most parents (60%) also reported difficulties with their child understanding instructions, fear or stress with eye drops and communicating with staff. A complete breakdown of reported barriers is presented in supplemental material Table S1.

Participants were asked which supports would help make eye examinations and related services more accessible and comfortable. Most autistic adults (81%) and parents (90%) indicated that having eye care staff who were knowledgeable and understanding of autism was a key enabler. A calm and reassuring examination environment was also valued (76% of autistic adults; 88% of parents), alongside clear and straight forward instructions (70% of adults; 87% of parents) and adjustments to the sensory environment (74% of adults; 73% of parents). When asked which methods of communication and information delivery were most important to them during eye care services, the most frequently endorsed preference by autistic adults was receiving digital reminders and instructions (71%), while parents highlighted the importance of visual aids (73%). Most autistic adults (61%) and parents (71%) emphasised the importance of plain language communication. A full breakdown of valued supports is presented in supplemental material Table S2.

### Qualitative results

**Figure 1.**
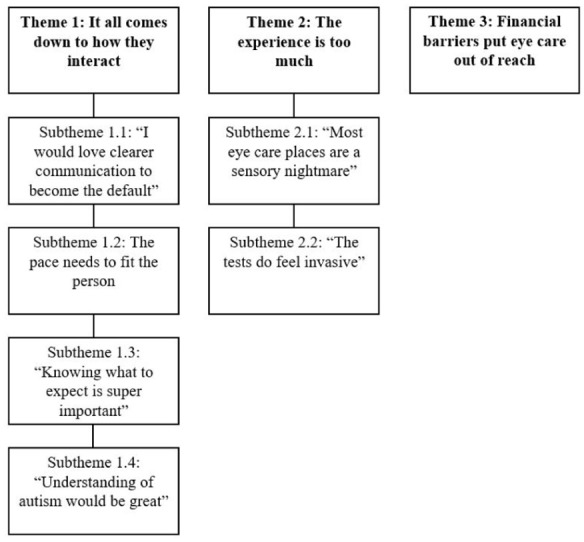
Overview of themes reflecting eye care experiences.

Participants were invited to share open-ended reflections on their experiences with eye care and suggestions for improving services. These qualitative responses were analysed thematically to complement the quantitative findings and to foreground the lived experiences behind the broader patterns observed. Three overarching themes were developed, with the third primarily being described by autistic adults: (1) it all comes down to how they interact, (2) the experience is too much, and (3) financial barriers put eye care out of reach. A visual summary of these themes and their corresponding subthemes is presented in Figure 1. Participant quotes are attributed using identifiers, with ‘AA’ denoting autistic adults and ‘P’ denoting parents or caregivers (e.g. AA-19, P-55).

#### Theme 1: it all comes down to how they interact

This theme captures how the quality of interpersonal interactions, particularly communication style, pacing, clarity, and understanding of autism, influenced participants’ overall experiences of eye care. These interactions often determined whether the experience felt supportive and accessible or confusing and distressing.

##### Subtheme 1.1: ‘I would love clearer communication to become the default’

Participants emphasised that unclear or overly medicalised terminology used during eye care appointments often led to confusion, anxiety, and reduced engagement. One autistic adult explained that ‘it’s very medical and there isn’t much talking about what’s happening’ (AA-62), while another simply wanted eye care professionals to ‘please stop using medical jargon’ (AA-179). Parents echoed these concerns, describing ‘a lack of communication skills from professionals’ (P-169) and distress when their children could not follow ‘the descriptions given by the optometrist’ (P-152).

Vague or ambiguous instructions, particularly during lens comparison tasks (known clinically as subjective refraction, where patients are asked to choose which lenses make their vision clearer), were a common source of stress. One autistic adult recounted having ‘difficulty understanding what they meant when clarifying which things are more in focus . . . it didn’t make sense’ (AA-21). A parent noted the need to repeatedly ‘explain everything to my son in easier to understand language’ (P-28). In response, participants called for the use of ‘plain English, especially around instructions’ (P-148), more descriptive guidance, and communication styles attuned to different processing and interpretation needs.

##### Subtheme 1.2: the pace needs to fit the person

Participants described how the pace of appointments, particularly vision assessments and choosing frames, influenced their comfort, confidence, and overall experience. Being rushed made it difficult to think clearly or communicate needs, whereas being given time and space fostered trust and reduced stress. One parent expressed feeling like ‘a burden on them because the appointment may stretch out a little longer’ (P-141), while an autistic adult shared, ‘I really struggle when they’re rushing me. To choose a frame, or to get tested. It’s pretty awful’ (AA-6). Time pressure during lens comparison tasks was a particular source of stress, with one parent describing their child not being ‘given time [when comparing] between lenses’ (P-55). Another participant explained that ‘just because I’m slow to respond doesn’t mean I didn’t hear or don’t understand . . . I’m constantly feeling rushed during my appointment’ (AA-125), and others worried that slow responses might be ‘assumed to mean I was making things up’ (AA-81).

Despite these challenges, some participants described positive experiences when staff were attuned to the pace of the appointment, allowing more flexible timing when needed. One recalled when ‘they asked if I was ok with the speed of which they were going’ (AA-179), while another shared how ‘the person helping me allowed me to sit alone in a darkened room to self-regulate before moving on to the next task when I was ready’ (AA-41). Suggestions such as ‘booking a longer session so that time can be taken’ (P-55) highlight the importance of pacing and appreciation of patient needs in creating a more accessible and respectful care experience.

##### Subtheme 1.3: ‘knowing what to expect is super important’

Participants highlighted how a lack of clear, upfront information about appointments contributed significantly to anxiety and, in some cases, avoidance of eye care. Several autistic adults described feeling unprepared for procedures, ‘often during an eye exam, they don’t tell you what’s coming – bright lights etc. They also don’t tell you what they’re testing for’ (AA-73). One person reflected on their experience with a more complex procedure, ‘I would really have appreciated something like a step-by-step guide with some photos, and information about what sensations to expect. . . I definitely considered delaying the procedure, because I really struggle with the unknowns’ (AA-107). In contrast, a positive experience was shared by a participant who feared the eye puff test (a measure of intra-ocular pressure) who recalled being supported by watching a demonstration of the procedure from the optometrist on their colleague, ‘knowing what the machine sounded like and the pressure was helpful . . . It made the pressure and the noise much less scary. . . I have not had a problem with the test since then’ (AA-21). Parents similarly emphasised the importance of preparation, often taking the lead themselves to reduce their child’s anxiety. Strategies included ‘googling videos of children at optometrists to watch together’ (P-55) and ‘explaining to my son before any medical appointment what is likely to happen’ (P-33).

##### Subtheme 1.4: ‘understanding of autism would be great’

Participants described a widespread lack of understanding of autism among eye care professionals and staff. In some instances, this led to insensitive or distressing interactions. One participant described a provider who was ‘very aggressive and rough with my son . . . she then got two other staff members to hold him down because she said that all autistic children are aggressive’, despite the parent describing their child as being ‘an extremely gentle and easygoing toddler’ (P-142). Others recalled staff who became ‘frustrated with my daughter as she couldn’t understand their instructions’ (P-15) or engaged in infantilising behaviour, such as ‘talking to me like a child’ (AA-179). These experiences impacted participants’ willingness to return for future care, ‘I just avoid going as I have found there to be no understanding of my needs’ (AA-101). Another noted, ‘if optometrists were more knowledgeable and understanding of my conditions, I might go to them, but I haven’t had any positive experiences’ (AA-153).

In response to these experiences, participants repeatedly called for systemic changes in staff training and professional development. One parent described the lack of autism knowledge as ‘unacceptable . . . rather than think outside the box to help my child, it’s thrown in the too hard basket’ (P-141). Another participant explained, ‘it’s almost completely inaccessible for me because I don’t have anywhere I can go that guarantees even a basic understanding of being autistic’ (AA-26). Calls for training extended beyond clinicians to administrative and shop floor staff, ‘better training in understanding neurodiversity for front office staff—optometrists are usually fine, but everyone else can make things harder’ (AA-24). Others urged a deeper, more values-based shift, ‘I think it’s important that healthcare professionals are trained and open-minded that they need to make accommodations and adjustments. Patience is also key’ (AA-23), and, more simply, ‘a true understanding of autism and neurodiversity’ (AA-187).

#### Theme 2: the experience is too much

##### Subtheme 2.1: ‘most eye care places are a sensory nightmare’

Participants described eye care settings as profoundly overwhelming, often triggering distress or shutdown. Many participants noted that ‘shopping centres aren’t very autism friendly’ (AA-19), yet, frustratingly, most optometry clinics were located within them. As one autistic adult shared, ‘I can’t access shopping centres anymore. I don’t know where an optometrist is that isn’t in a shopping centre’ (AA-19). For others, the cumulative burden of navigating traffic, waiting rooms, and clinical procedures meant that ‘meltdowns, safety issues and cancellation of appointments’ became common outcomes when trying to attend a scheduled eyecare appointment (P-157). Sensory aspects of the environment including ‘noise and bright lights’ (P-28), ‘so many people in the waiting room’ (AA-52), and the general ‘busy, loud and bright’ (AA-82) nature of clinics, were frequently cited as barriers. One parent described having to ‘go and wait in our car’ due to the absence of a suitable indoor waiting space. Another autistic adult explained:

The environment is my main barrier . . . I have to split up appointments and go back at different times for different stages so I don’t get overwhelmed. The last time I got new glasses it took four different appointments to arrange them . . . I wish there had been an option to have some appointments in a quieter environment. (AA-82)

Participants expressed a clear desire for low-stimulation spaces, including ‘the ability to relax in a sensory room or quiet space by ourselves’ (P-47). In anticipation of these challenges, participants described implementing personal strategies, such as ‘scheduling time after the appointment to recover’ (AA-19) or ‘spending the rest of the day in low lighting’ (AA-157), highlighting the effort required to simply attend an appointment in the absence of sensory-accessible care.

##### Subtheme 2.2: ‘the tests do feel invasive’

While the broader sensory context of eye care was often challenging, participants identified particular clinical procedures as especially ‘difficult for a lot of us’ (AA-23). Among these was an eye pressure test, which several participants described as intolerable. One autistic adult explained, ‘I just tell them not to do it as it is too stressful and distressing for me’ (AA-4). Others described intense discomfort with ‘the bright light that makes me almost pass out because I’m super sensitive to light’ (AA-26). As one participant summarised, ‘the whole experience hurts my eyes and is overwhelming. I’m more likely to see a dentist than an optometrist’ (AA-153).

Several participants highlighted the difficulty of maintaining stillness during procedures, reporting that ‘you’re instructed to sit still and it’s difficult for me to do this’ (AA-23). Small, dark consulting rooms also contributed to distress and sensory overload. As one autistic adult described, ‘the coldness of the machine, the dark (and unusually tiny) room. . . even the shifts between the different lens can cause my brain to do flips’ (AA-125). Others noted that ‘it’s a very intimate/close proximity to a stranger in a small space’, which added to the discomfort (AA-26).

Parents similarly described distress related to specific procedures (e.g. eye pressure test), especially when practitioners did not adjust their approach in response to visible signs of anxiety. One parent recalled an appointment where the optometrist ‘continued to attempt to do [the eye pressure test] despite the child cowering and crying’ (P-155) Others noted that procedures involving drops, lights, or unfamiliar equipment could be ‘painful’, (P-156), ‘scary’ (P-146) or simply too much, ‘I don’t want to put him through a test which will be distressing if there is nothing wrong’ (P-119).

#### Theme 3: financial barriers put eye care out of reach

Some autistic adults described the cost of eye care, particularly prescription glasses and specialist appointments, as a significant barrier to accessing services. While one acknowledged that ‘it’s great that we can get free eye checks through Medicare^2^’ (AA–19), many still avoided appointments because ‘I couldn’t afford glasses anyway. So why bother having an eye test?’ (AA–6). Other participants described financial insecurity as a structural barrier to accessing eye care. As an autistic adult explained, ‘autistic people have a much lower income stream on average. This is a huge factor in preventing me from accessing proper eye care’ (AA–6). Another similarly noted, ‘we often work less, have less money, can’t afford private healthcare, and have to pay the same price as everyone else for glasses’ (AA–73). These financial barriers led some to delay or entirely forgo necessary care, ‘I have delayed seeking eye care because of financial constraints’ (AA–30) and ‘I need new glasses but can’t afford to buy them so there’s no point in an eye test’ (AA–67). For others, cost prohibited access to specialist care, ‘I was referred by the ENT [Ear Nose Throat] emergency hospital to an ophthalmologist but could not afford the fees’ (AA–128).

## Discussion

This study sheds new light on the experiences of autistic adults and families accessing eye care in Australia and Aotearoa New Zealand. Participants described multiple, interrelated barriers that undermined their, or their child’s, ability to engage with services, including unclear communication, sensory discomfort, anxiety, financial strain, and limited staff understanding of autism. At the same time, they identified simple but meaningful adjustments, such as plain language, flexible pacing, and advance preparation as helpful in enabling more accessible, respectful, and affirming care.

These findings align closely with those of Parmar et al. (2022), who identified similar access challenges among autistic adults in the United Kingdom, including sensory overload, unpredictable processes, and stress associated with unfamiliar staff interactions. As in that study, participants here reported difficulties across every stage of the eye care journey, from making appointments to navigating the waiting room, undergoing testing, and interacting with providers. This study extends this work in several important ways. First, by including the perspectives of both autistic adults and parents, the study was able to examine eye care experiences across a broader range of age groups and support needs, offering a more comprehensive understanding of access barriers and enablers across the life span. Second, its mixed-methods design allows for deeper insight into both the extent and lived experience of barriers. Finally, participants in this study drew attention to systemic factors, such as the cost of glasses and the sensory environments of shopping centre-based clinics, that compounded difficulties in accessing care.

While earlier studies (Coulter et al., 2015; Little, 2018; Parmar et al., 2022) have highlighted the value of adjustments made at service level, our findings suggest that these changes, while important, are not sufficient. Access challenges were often structural and cumulative, pointing to the need for more coordinated, system-level responses. This supports growing calls for healthcare reform across patient, provider, and system levels (Babalola et al., 2024; Hamdan & Bennett, 2024; Walsh et al., 2020) and suggests that improving access will require more than ad hoc accommodations. These findings also resonate with emerging models of autism-informed healthcare that advocate for systemic, rather than piecemeal approaches to accessibility. While this study is grounded in the specific experiences of autistic people and their families, future research could explore how autism-affirming practices align with, or challenge, broader frameworks such as universal design. Such exploration may inform more inclusive healthcare models while ensuring autistic needs are still met.

For example, the Autistic SPACE framework (Doherty et al., 2023) outlines five domains, Sensory, Predictability, Acceptance, Communication, and Empathy, which collectively define the foundations of accessible and respectful care. Although these adjustments may appear straightforward, implementing them consistently requires services to rethink how care is delivered, particularly in the face of broader structural barriers such as financial constraints and overstimulating clinical environments. This model, alongside related work by O’Hagan et al. (2023) and Hamdan and Bennett (2024), emphasises that supports such as reducing sensory stress, providing clear information, and increasing predictability require a shift in system design and professional culture. Rather than relying on reactive, case-by-case accommodations, services must adopt a proactive, embedded approach that anticipates and supports the diverse needs of autistic people from the outset. Encouragingly, practical resources are beginning to reflect these principles in action, for example, the University of Manchester has developed autism-informed materials for both patients and providers, designed to support more accessible and affirming eye care interactions (University of Manchester, n.d.).

One of the most pressing systemic issues identified in this study was financial exclusion. Although routine vision testing is publicly funded in Australia through Medicare, some participants reported avoiding appointments because they could not afford glasses, contact lenses or specialist care. This is understandable, given that many autistic adults report low income and poor financial wellbeing (Cai et al., 2023, 2024; Pellicano, Hall, & Ying Cai, 2024). This financial disadvantage is often a consequence of high rates of underemployment and poor job-matching in Australia (Australian Bureau of Statistics, 2024; Harvery et al., 2021). Similar patterns of economic insecurity among autistic adults have been reported internationally (Davies et al., 2024; Vincent & Ralston, 2024), underscoring the compounding effect of employment inequity on healthcare access. Without coordinated action across employment and health systems, access to essential services like eye care will remain inequitable. In countries lacking universal healthcare coverage, these intersecting disadvantages are likely to be even more pronounced.

Taken together, these findings highlight the urgent need for cross-sector solutions that address barriers at individual, organisational, and policy levels. While sensory and communication adjustments are essential, they must be embedded within broader strategies that also address affordability, access pathways, and continuity of care. Most importantly, these changes must be designed in partnership with autistic people and families, whose lived expertise is crucial for building services that are not only effective, but also safe, affirming, and genuinely inclusive.

### Strengths and limitations

This study was strengthened by the diverse expertise of its multidisciplinary international research team, which included an autistic researcher, autism researchers, family members of autistic people, and professionals in ophthalmology, optometry, and orthoptics. This combination of lived experience, clinical insight, and autism scholarship shaped the study design, analysis, and interpretation. The mixed-methods approach enabled both breadth and depth as quantitative data provided a broad overview of barriers and enablers, while qualitative findings offered detailed insights into how these issues are experienced in everyday life. Including both autistic adults and parents allowed exploration of overlapping but distinct perspectives on navigating care.

Nonetheless, the study has limitations. The sample lacked demographic diversity, with most participants identifying as White and residing in Australia; responses from Aotearoa New Zealand and from parents were limited. Recruitment via autism networks may have led to overrepresentation of people with greater engagement or more direct experience of challenges in eye care. The online survey format likely excluded autistic people with intellectual disability or high support needs, groups whose experiences may differ substantially and remain underrepresented in research (Gibbs et al., 2024; Russell et al., 2019) and may also have limited the depth of individual responses compared to more interactive qualitative methods. Although the study defined ‘eye care’ broadly, most responses focused on routine optometry rather than specialist or hospital-based care. Future research should examine the experiences of autistic people across a wider range of eye health services, particularly in underserved populations such as Indigenous peoples, people with complex communication needs, and those in rural or remote areas.

## Conclusion

This study highlights the persistent barriers that autistic people and their families face in accessing equitable, respectful eye care. These include sensory and communication challenges, financial constraints, and a lack of autism understanding in many service settings. Participants also identified simple, practical adjustments, such as clearer communication, more predictable processes, and sensitivity to sensory needs, that could make a significant difference. However, achieving true equity will require more than individual goodwill or minor adjustments; it demands systemic change in how services are organised, delivered, and funded. Importantly, these changes must be developed in partnership with autistic people, to ensure that services are not only clinically effective but genuinely inclusive, responsive, and affirming of neurodivergent ways of being.

## Supplemental Material

sj-docx-1-aut-10.1177_13623613251371509 – Supplemental material for Understanding eye care access for autistic adults and families: A convergent mixed-methods study Supplemental material, sj-docx-1-aut-10.1177_13623613251371509 for Understanding eye care access for autistic adults and families: A convergent mixed-methods study by Chris Edwards, Abigail MA Love, Ru Ying Cai, Paul Constable, Daniel C Love, Ketan Parmar, Emma Gowen and Vicki Gibbs in Autism

sj-docx-2-aut-10.1177_13623613251371509 – Supplemental material for Understanding eye care access for autistic adults and families: A convergent mixed-methods study Supplemental material, sj-docx-2-aut-10.1177_13623613251371509 for Understanding eye care access for autistic adults and families: A convergent mixed-methods study by Chris Edwards, Abigail MA Love, Ru Ying Cai, Paul Constable, Daniel C Love, Ketan Parmar, Emma Gowen and Vicki Gibbs in Autism

sj-docx-3-aut-10.1177_13623613251371509 – Supplemental material for Understanding eye care access for autistic adults and families: A convergent mixed-methods study Supplemental material, sj-docx-3-aut-10.1177_13623613251371509 for Understanding eye care access for autistic adults and families: A convergent mixed-methods study by Chris Edwards, Abigail MA Love, Ru Ying Cai, Paul Constable, Daniel C Love, Ketan Parmar, Emma Gowen and Vicki Gibbs in Autism

## Notes

1. This inclusive approach recognises the range of barriers that impede access to autism assessment and diagnosis (Ardeleanu et al., 2024).

2. Medicare is a publicly funded universal healthcare insurance scheme in Australia.

## References

[bibr1-13623613251371509] Ardeleanu K. Steinberg H. Garfield T. Voltaire S. Shea L. Brown M. Chvasta K. Tan C. D. (2024). Self-identification of autism: Why some autistic adults lack a clinical diagnosis and why this matters for inclusion. Autism, 29(9), 2344–2355. 10.1177/13623613241297222 39552426

[bibr2-13623613251371509] Australian Bureau of Statistics. (2024). Autism in Australia, 2022. https://www.abs.gov.au/articles/autism-australia-2022

[bibr3-13623613251371509] Babalola T. Sanguedolce G. Dipper L. Botting N. (2024). Barriers and facilitators of healthcare access for autistic children in the UK: A systematic review. Review Journal of Autism and Developmental Disorders. Advance online publication. 10.1007/s40489-023-00420-3

[bibr4-13623613251371509] Braun V. Clarke V. (2006). Using thematic analysis in psychology. Qualitative Research in Psychology, 3(2), 77–101. 10.1191/1478088706QP063OA

[bibr5-13623613251371509] Braun V. Clarke V. Hayfield N. Terry G. (2019). Thematic analysis. In Liamputtong P. (Ed.), Handbook of research methods in health social sciences (pp. 843–860). Springer. 10.1007/978-981-10-5251-4_103

[bibr6-13623613251371509] Cai R. Y. Gallagher E. Haas K. Love A. Gibbs V. (2023). Exploring the income, savings and debt levels of autistic adults living in Australia. Advances in Autism, 9(1), 53–64. 10.1108/AIA-01-2022-0004

[bibr7-13623613251371509] Cai R. Y. Hall G. Pellicano E. (2024). Predicting the financial wellbeing of autistic adults: Part I. Autism, 28(5), 1203–1215. 10.1177/13623613231196085 37665058

[bibr8-13623613251371509] Calleja S. Islam F. M. A. Kingsley J. McDonald R. (2020). Healthcare access for autistic adults: A systematic review. Medicine, 99(29), Article e20899. 10.1097/MD.0000000000020899 PMC7373620 32702830

[bibr9-13623613251371509] Corden K. Brewer R. Cage E. (2022). A systematic review of healthcare professionals’ knowledge, self-efficacy and attitudes towards working with autistic people. Review Journal of Autism and Developmental Disorders, 9(3), 386–399. 10.1007/s40489-021-00263-w

[bibr10-13623613251371509] Coulter R. A. Bade A. Tea Y. Fecho G. Amster D. Jenewein E. Rodena J. Lyons K. K. Mitchell G. L. Quint N. Dunbar S. Ricamato M. Trocchio J. Kabat B. Garcia C. Radik I. (2015). Eye examination testability in children with autism and in typical peers. Optometry and Vision Science, 92(1), 31–43. 10.1097/OPX.0000000000000442 25415280 PMC4274340

[bibr11-13623613251371509] Creswell J. W. Plano Clark V. L. (2018). Designing and conducting mixed methods research (3rd ed.). SAGE.

[bibr12-13623613251371509] Davies J. Romualdez A. M. Pellicano E. Remington A. (2024). Career progression for autistic people: A scoping review. Autism, 28(11), 2690–2706. 10.1177/13623613241236110 38477466 PMC11494842

[bibr13-13623613251371509] Doherty M. McCowan S. Shaw S. C. K. (2023). Autistic SPACE: A novel framework for meeting the needs of autistic people in healthcare settings. British Journal of Hospital Medicine, 84(4), 1–9. 10.12968/hmed.2023.0006 37127416

[bibr14-13623613251371509] Edwards C. Love A. M. A. Cai R. Y. Tutton T. Beardsley E. Gibbs V. (2025). Autistic-led insights on airport accessibility: A retrospective analysis of environmental assessments. Autism, 29(8), 2151–2162. 10.1177/13623613251337200 40340572 PMC12255834

[bibr15-13623613251371509] Gibbs V. Cai R. Y. Love A. Edwards C. Al Ansari M. (2024). Unheard voices: A systematic literature review of studies using self-report methods to gather the perspectives of autistic adults with intellectual disability. Autism in Adulthood. Advance online publication. 10.1089/aut.2024.0272 PMC13315303 42383261

[bibr16-13623613251371509] Hamdan S. Z. Bennett A. (2024). Autism-friendly healthcare: A narrative review of the literature. Cureus, 16(7), Article e64108. 10.7759/cureus.64108 PMC11305600 39114203

[bibr17-13623613251371509] Harvery M. Froude E. H. Foley K. R. Trollor J. N. Arnold S. R. (2021). Employment profiles of autistic adults in Australia. Autism Research, 14(10), 2061–2077. 10.1002/aur.2588 34374491

[bibr18-13623613251371509] Lindly O. J. Chan J. Fenning R. M. Farmer J. G. Neumeyer A. M. Wang P. Swanson M. Parker R. A. Kuhlthau K. A. (2021). Vision care among school-aged children with autism spectrum disorder in North America: Findings from the autism treatment network registry call-back study. Autism, 25(3), 840–853. 10.1177/1362361320942091 32693628

[bibr19-13623613251371509] Little J.-A. (2018). Vision in children with autism spectrum disorder: A critical review. Clinical & Experimental Optometry, 101(4), 504–513. 10.1111/cxo.12651 29323426

[bibr20-13623613251371509] Mason D. Stewart G. R. Capp S. J. Happé F. (2022). Older age autism research: A rapidly growing field, but still a long way to go. Autism in Adulthood, 4(2), 164–172. 10.1089/aut.2021.0041 36605971 PMC9645679

[bibr21-13623613251371509] Nadeem Z. A. Akram U. Khalid T. B. Nadir M. A. Akhtar M. H. (2024). Refractive errors linked to autism spectrum disorders in the pediatric population and young adults: A systematic review and meta-analysis. Review Journal of Autism and Developmental Disorders. Advance online publication. 10.1007/s40489-024-00468-9

[bibr22-13623613251371509] O’Hagan B. Krauss S. B. Friedman A. J. Bartolotti L. Abubakare O. Broder-Fingert S. Augustyn M. (2023). Identifying components of autism friendly health care: An exploratory study using a modified Delphi method. Journal of Developmental & Behavioral Pediatrics, 44(1), e12–e18. 10.1097/DBP.0000000000001139 36367772

[bibr23-13623613251371509] Parmar K. R. Porter C. S. Dickinson C. M. Baimbridge P. Pelham J. Gowen E. (2022). Autism-friendly eyecare: Developing recommendations for service providers based on the experiences of autistic adults. Ophthalmic & Physiological Optics, 42(4), 675–693. 10.1111/opo.12975 35315935 PMC9313607

[bibr24-13623613251371509] Pellicano E. Adams D. Crane L. Hollingue C. Allen C. Almendinger K. Botha M. Haar T. Kapp S. K. Wheeley E. (2024). Letter to the Editor: A possible threat to data integrity for online qualitative autism research. Autism, 28(3), 786–792. 10.1177/13623613231174543 37212144

[bibr25-13623613251371509] Pellicano E. Dinsmore A. Charman T. (2014). What should autism research focus upon? Community views and priorities from the United Kingdom. Autism, 18(7), 756–770. 10.1177/1362361314529627 24789871 PMC4230972

[bibr26-13623613251371509] Pellicano E. Hall G. Ying Cai R. (2024). Autistic adults’ experiences of financial wellbeing: Part II. Autism, 28(5), 1090–1106. 10.1177/13623613231191594 37795595 PMC11067415

[bibr27-13623613251371509] Perna J. Bellato A. Ganapathy P. S. Solmi M. Zampieri A. Faraone S. V. Cortese S. (2023). Association between autism spectrum disorder (ASD) and vision problems. A systematic review and meta-analysis. Molecular Psychiatry, 28(12), 5011–5023. 10.1038/s41380-023-02143-7 37495888

[bibr28-13623613251371509] Radev S. Freeth M. Thompson A. R. (2024). How healthcare systems are experienced by autistic adults in the United Kingdom: A meta-ethnography. Autism, 28(9), 2166–2178. 10.1177/13623613241235531 38465626 PMC11403927

[bibr29-13623613251371509] Reynolds M. Culican S. M. (2023). Visual autism. Children, 10(4), 606. 10.3390/children10040606 37189855 PMC10136985

[bibr30-13623613251371509] Russell G. Mandy W. Elliott D. White R. Pittwood T. Ford T. (2019). Selection bias on intellectual ability in autism research: A cross-sectional review and meta-analysis. Molecular Autism, 10, 1–10. 10.1186/s13229-019-0260-x 30867896 PMC6397505

[bibr31-13623613251371509] Shaw S. C. Carravallah L. Johnson M. O’Sullivan J. Chown N. Neilson S. Doherty M. (2024). Barriers to healthcare and a ‘triple empathy problem’ may lead to adverse outcomes for autistic adults: A qualitative study. Autism, 28(7), 1746–1757. 10.1177/13623613231205629 37846479 PMC11191657

[bibr32-13623613251371509] Swanson M. W. Lee S. D. Frazier M. G. Bade A. Coulter R. A. (2020). Vision screening among children with autism spectrum disorder. Optometry and Vision Science, 97(11), 917–928. 10.1097/OPX.0000000000001593 33136709

[bibr33-13623613251371509] University of Manchester. (n.d.). Autism, vision and eyecare. https://sites.manchester.ac.uk/autism-and-vision/

[bibr34-13623613251371509] Vincent J. Ralston K. (2024). Uncovering employment outcomes for autistic university graduates in the United Kingdom: An analysis of population data. Autism, 28(3), 732–743. 10.1177/13623613231182756 37353923 PMC10913337

[bibr35-13623613251371509] Walsh C. Lydon S. O’Dowd E. O’Connor P. (2020). Barriers to healthcare for persons with autism: A systematic review of the literature and development of a taxonomy. Developmental Neurorehabilitation, 23(7), 413–430. 10.1080/17518423.2020.1716868 36112897

[bibr36-13623613251371509] Weir E. Allison C. Baron-Cohen S. (2022). Autistic adults have poorer quality healthcare and worse health based on self-report data. Molecular Autism, 13, 23. 10.1186/s13229-022-00501-w 35619147 PMC9135388

[bibr37-13623613251371509] Wu C.-S. Tsai T.-H. Chen W.-L. Tsai H.-J. Chien Y.-L. (2023). Ophthalmologic diagnoses in youths with autism spectrum disorder: Prevalence and clinical correlates. Autism Research, 16(10), 2008–2020. 10.1002/aur.3019 37632715

