# Supplementary material for: Understanding eye care access for autistic adults and families: A convergent mixed-methods study
Source: Autism. 2025 Sep 20;30(1):122–33. doi: 10.1177/13623613251371509 (PMC12717288; doi:10.1177/13623613251371509)
Supplement: sj-docx-1-aut-10.1177_13623613251371509 – Supplemental material for Understanding eye care access for autistic adults and families: A convergent mixed-methods study [file sj-docx-1-aut-10.1177_13623613251371509.docx]

[insert participant information sheet and consent]

**Block 1: Suitability**

1. Do you live in Australia or New Zealand?
   - Yes (Australia)
   - Yes (New Zealand)
   - No -> ineligible
2. Are you aged 18 years or older?
   - Yes
   - No -> ineligible
3. From which perspective will you be completing this survey? (Please select one)
   - I am an Autistic adult
   - I am the parent/caregiver of an Autistic person
   - I am neither of the above -> ineligible

*Note: If you identify as both an Autistic adult and a parent/caregiver of an Autistic person OR have multiple Autistic children, you can complete the survey more than once.*

Thank you for your interest in our survey exploring the eye care experiences of Autistic people. Unfortunately, your response(s) indicates this survey is not suitable for you. This survey is for Autistic adults and parents of an Autistic person who live in Australia or New Zealand.

**Block 2: Demographics**

The following questions will help us understand the background of our participants, allowing us to consider the diverse experiences in our analysis.

1. What is your age in years? (e.g. 30)
   - [text]
2. What gender do you identify with?
   - Female
   - Male
   - Non-binary
   - Other (please specify): [text]
   - Rather not say
3. What is your current relationship status?
   - Single
   - In a de facto relationship
   - Married
   - Other (please specify): [text]
   - Rather not say
4. What best describes your ethnicity? (please select all that apply)
   - Aboriginal Australian
   - Torres Strait Islander
   - Māori
   - Pacific Islander
   - South Asian
   - South East Asian
   - Middle Eastern
   - African
   - Latin American/Hispanic
   - European/White
   - Mixed heritage
   - Other (please specify): [text]
   - Prefer not to say
5. What is the highest level of education you have completed?
   - Primary school
   - High school
   - Vocational education and training (e.g. TAFE)
   - Higher education (e.g. university)
   - Other (please specify): [text]
   - Rather not say
6. What employment category currently best describes you?
   - Employed: full time
   - Employed: part time/casual
   - Self-employed
   - Unemployed
   - Retired
   - Other (please specify): [text]
   - Rather not say

**Block 3A: Autistic Adult Questions**

1. What best describes your Autism diagnosis/identity?
   - I have received a formal Autism diagnosis
   - I have not received a formal diagnosis but I self-identify as Autistic
2. Do you have any other conditions?
   - Yes
   - No
   - I don’t know
3. If Yes: Please select all that apply to you (there is a separate question about eye conditions, so you do not need to include those under ‘Other’ here)
   - ADHD/ADD
   - Intellectual Disability
   - Anxiety Disorder (e.g. social anxiety disorder)
   - Mood Disorder (e.g. depression)
   - Personality Disorder
   - Sleep Disorder (e.g. insomnia)
   - Eating Disorder
   - Other (please specify): [text]
   - Rather not say/not sure

**Block 3B: Parent/Caregiver Questions**

1. What is your Autistic child’s age in years?
   - [text]
2. What gender do they identify with?
   - Male
   - Female
   - Non-binary
   - Transgender
   - Other (please specify): [text]
   - Rather not say/not sure
3. What best describes their ethnicity? (please select all that apply)
   - Aboriginal Australian
   - Torres Strait Islander
   - Māori
   - Pacific Islander
   - South Asian
   - South East Asian
   - Middle Eastern
   - African
   - Latin American/Hispanic
   - European/White
   - Mixed heritage
   - Other (please specify): [Text]
   - Rather not say/not sure
4. Have they received a formal Autism diagnosis?
   - Yes
   - No but they self-identify as Autistic
5. Do they have any other conditions?
   - Yes
   - No
6. If Yes: Please select all which are applicable for them (there is a separate question about eye conditions coming up, so you do not need to include those under ‘Other’ here)
   - ADHD/ADD
   - Intellectual Disability
   - Anxiety Disorder (e.g. social anxiety disorder)
   - Mood Disorder (e.g. depression)
   - Personality Disorder
   - Sleep Disorder (e.g. insomnia)
   - Eating Disorder
   - Other (please specify): [text]
   - Rather not say/not sure

**Block 4A: Eye care context questions**

In this section, we will ask about your experiences with eye care, which includes routine eye exams, management of eye conditions, and the types of eye care professionals you or your child may have seen.

1. How often do you (or your child) have an eye exam? (provide your best guess)
   - About every year
   - About every 2 years
   - Less frequently
   - Never had an eye exam
   - Other (please specify): [text]
2. Was there a time when you (or your child) wanted to visit an eye care professional but couldn’t?
   - Yes
   - No
   - Not sure/I don’t remember
3. If Yes: Can you explain why you couldn’t access an eye care service when it was needed?
   - [text]
4. What type of eye care professional have you (or your child) seen before? (please select all that apply)
   - Optometrist (primary eye care provider)
   - Ophthalmologist (medical doctor specialising in eye and vision care)
   - Orthoptist (specialist in diagnosing and treating eye movement disorders)
   - Not sure
   - Never seen an eye care professional
5. What type of eye care services have you (or your child) used in the past? (please select all that apply)
   - Routine eye exams (regular vision checks)
   - Prescription of glasses or contact lenses (corrective lenses)
   - Management of eye conditions (e.g., eye infection such as conjunctivitis)
   - Vision therapy (exercises and therapies to improve visual skills)
   - Low vision services (support for significant vision impairment)
   - Paediatric eye care (eye care specifically for children)
   - Occlusion therapy (e.g. patching for lazy eye)
   - Emergency eye care (urgent treatment for eye problems and injuries)
   - Other (please specify): [text]
6. Have you (or your child) been diagnosed with any of the following eye conditions? (please select all that apply)
   - Amblyopia (lazy eye)
   - Strabismus (crossed eyes)
   - Refractive errors (e.g., myopia, hyperopia, astigmatism) (vision correction needs)
   - Cataracts (clouding of the eye lens)
   - Glaucoma (optic nerve damage)
   - Retinoblastoma (eye tumour)
   - Retinopathy of prematurity (eye disease in babies who are premature)
   - Other (please specify): [text]
   - None/I don’t know

**Block 4B: Eye care barrier/challenges questions**

In this section, we would like to understand any challenges or difficulties you (or your child) may face during eye exams, accessing eye care services, or obtaining information related to these services. Your responses will help identify specific barriers, whether related to autism, other conditions, or general circumstances.

1. Do you (or your child) experience any difficulties during eye exams, which include routine checks of vision and eye health conducted by eye care professionals? (please select all that apply)
   - Sensory sensitivities (e.g. discomfort with bright lights near the eye, being in a dark room with a flashlight shining)
   - Feelings of anxiety or stress
   - Difficulty understanding instructions
   - Communication differences (e.g. difficulty communicating with the eye care professional during assessments)
   - Difficulty remaining still
   - Unclear explanations of the process
   - Sudden changes or unexpected events
   - Fear or stress with eye drops
   - Unwillingness to try on or wear glasses
   - Challenges in assessing level of vision
   - Other (please specify): [text]
   - No difficulties
2. Do you (or your child) experience any difficulties when accessing eye care services, such as scheduling appointments, visiting the clinic, or obtaining glasses or treatments? (please select all that apply)
   - Difficulty scheduling appointments
   - Long waiting times
   - Inadequate communication from providers
   - Lack of understanding of specific needs by eye care staff (e.g., autism, other conditions)
   - Uncomfortable sensory environment
   - Multiple staff interactions causing anxiety
   - Unfamiliar or changing practice layout
   - Pressure to make quick decisions (e.g. choosing glasses)
   - Physical accessibility issues (e.g. getting to the clinic)
   - Financial costs (e.g. cost of exams, glasses, or treatment)
   - Other (please specify): [text]
   - No challenges
3. Please feel free to expand on what you think makes it hard for Autistic people to access eye care services?
   - [text]
4. If you have avoided or delayed seeking eye care, what specific concerns or experiences contributed to this decision?
   - [text]

**Block 4C: Eye care enabler/support questions**

In this section, we want to learn about the factors that can enhance your (or your child’s) experience with eye care services, as well as your suggestions for improvement.

1. What would help you/them have a more positive experience when accessing eye care services, including during eye exams? (please select all that apply)
   - Adjustments to lights and sounds that are sensitive to sensory needs
   - A calm and reassuring environment
   - Clear and straightforward instructions
   - Communication that is tailored to individual preferences and needs
   - Opportunities to take breaks when needed
   - Consistent interactions with familiar staff members
   - Use of visual aids or demonstrations
   - Information provided in advance about what to expect (e.g., videos, pictures)
   - Flexible and easy appointment scheduling
   - Minimising waiting times
   - Eye care staff with knowledge and understanding of autism
   - Time allowed to become familiar with the environment
   - Other (please specify): [text]
2. What methods of communication and information delivery during eye care services are important for you/them? (please select all that apply)
   - Written materials provided in accessible formats
   - Visual aids, such as diagrams or videos
   - Verbal explanations supported by visual materials
   - Digital reminders and instructions (e.g., emails, text messages)
   - In-person explanations accompanied by demonstrations
   - Plain language communication (clear, easy-to-understand language)
   - Other (please specify): [text]
3. Could you describe any positive experiences you (or your child) have had with eye care services that made accessing care easier?
   - [text]
4. What personal strategies or supports do you (or your child) use to help ensure a successful eye care appointment? Please share any approaches that have worked well for you.
   - [text]
5. What changes would you most like to see in eye care services to better meet the needs of Autistic individuals?
   - [text]
6. Is there anything else you would like to share about your (or your child’s) experiences with eye care?
   - [text]
