# Supplementary material for: Understanding eye care access for autistic adults and families: A convergent mixed-methods study
Source: Autism. 2025 Sep 20;30(1):122–33. doi: 10.1177/13623613251371509 (PMC12717288; doi:10.1177/13623613251371509)
Supplement: sj-docx-2-aut-10.1177_13623613251371509 – Supplemental material for Understanding eye care access for autistic adults and families: A convergent mixed-methods study [file sj-docx-2-aut-10.1177_13623613251371509.docx]

**Table S1**

*Barriers to Accessing Eye Care*

| **Barriers** | **Autistic adults**  **(self-report)** | **Autistic children**  **(parent-report)** |
| --- | --- | --- |
| Difficulties during eye exams^a^  Sensory sensitivities  Anxiety or stress  Understanding instructions  Remaining still  Fear or stress with eye drops  Communicating with staff  Assessing level of vision  Sudden changes or unexpected events  Unclear explanations of the process  Other  Unwillingness to try on or wear glasses  No difficulties | *n* = 113  94 (83%)  74 (65%)  38 (34%)  40 (35%)  34 (30%)  33 (29%)  41 (36%)  29 (26%)  28 (25%)  23 (20%)  11 (10%)  5 (4%) | *n* = 57  37 (65%)  44 (77%)  34 (60%)  28 (49%)  34 (60%)  34 (60%)  20 (35%)  20 (35%)  17 (30%)  7 (12%)  18 (32%)  3 (5%) |
| Difficulties when accessing eye care services^a^  Uncomfortable sensory environment  Pressure to make quick decisions  Financial costs  Multiple staff interactions causing anxiety  Lack of understanding of specific needs (e.g. autism)  Scheduling appointments  Long waiting times  Other  Inadequate communication from providers  Unfamiliar or changing practice layout  No difficulties  Physical accessibility of the service | *n* = 113  71 (63%)  69 (61%)  51 (45%)  45 (40%)  33 (29%)  35 (31%)  16 (14%)  18 (16%)  20 (18%)  15 (13%)  10 (9%)  12 (11%) | *n* = 57  26 (46%)  15 (26%)  19 (33%)  24 (42%)  28 (49%)  11 (19%)  19 (33%)  10 (18%)  7 (12%)  9 (16%)  12 (21%)  4 (7%) |

*Note.* ^a^Participants could select multiple options.
