# Supplementary material for: Understanding eye care access for autistic adults and families: A convergent mixed-methods study
Source: Autism. 2025 Sep 20;30(1):122–33. doi: 10.1177/13623613251371509 (PMC12717288; doi:10.1177/13623613251371509)
Supplement: sj-docx-3-aut-10.1177_13623613251371509 – Supplemental material for Understanding eye care access for autistic adults and families: A convergent mixed-methods study [file sj-docx-3-aut-10.1177_13623613251371509.docx]

**Table S2**

*Supports to Improve Access to Eye Care*

| **Enablers** | **Autistic adults**  **(self-report)** | **Autistic children**  **(parent-report)** |
| --- | --- | --- |
| Supports during eye exams^a^  Eye care staff with knowledge and understanding of autism  Calm and reassuring environment  Clear and straight forward instructions  Adjustments to sensory environment  Information provided in advance about what to expect  Communication tailored to individual preferences  Minimizing waiting times  Opportunities to take breaks  Flexible and easy appointment scheduling  Consistent interactions with familiar staff  Use of visual aids or demonstrations  Time allowed to become familiar with the environment  Other | *n* = 109  88 (81%)  83 (76%)  76 (70%)  81 (74%)  66 (61%)  56 (51%)  52 (48%)  54 (50%)  63 (58%)  50 (46%)  37 (34%)  32 (29%)  18 (17%) | *n* = 52  47 (90%)  46 (88%)  45 (87%)  38 (73%)  37 (71%)  39 (75%)  39 (75%)  36 (69%)  20 (38%)  31 (60%)  33 (64%)  31 (60%)  7 (13%) |
| Communication and information preferences^a^  Plain language communication  Verbal explanations supported by visual materials  Digital reminders and instructions  Visual aids  In-person explanations accompanied by demonstrations  Written materials provided in accessible formats  Other | *n* = 107  65 (61%)  67 (63%)  76 (71%)  43 (40%)  53 (50%)  46 (43%)  9 (8%) | *n* = 52  37 (71%)  32 (62%)  21 (40%)  38 (73%)  25 (48%)  19 (37%)  8 (15%) |

*Note.* ^a^Participants could select multiple options.
